# Dysfunction of Endocytic Kinase AAK1 in ALS

**DOI:** 10.3390/ijms151222918

**Published:** 2014-12-10

**Authors:** Bingxing Shi, Sean D. Conner, Jian Liu

**Affiliations:** 1 Department of Neuroscience, California Pacific Medical Center Research Institute, San Francisco, CA 94107, USA; E-Mail: bxsin@yahoo.com; 2 Department of Genetics, Cell Biology and Development, University of Minnesota, Minneapolis, MN 55455, USA; E-Mail: sdconner@umn.edu; 3 Department of Biological Sciences, Xi’an Jiaotong-Liverpool University, Suzhou 215123, China

**Keywords:** SOD1, ALS, AAK1, endocytosis, aggregates

## Abstract

Mechanisms of human mutant superoxide dismutase 1 (SOD1)-induced toxicity in causing the familial form of amyotrophic lateral sclerosis (ALS) remain elusive. Identification of new proteins that can selectively interact with mutant SOD1s and investigation of their potential roles in ALS are important to discover new pathways that are involved in disease pathology. Using the yeast two-hybrid system, we identified the adaptor-associated kinase 1 (AAK1), a regulatory protein in clathrin-coated vesicle endocytic pathway that selectively interacted with the mutant but not the wild-type SOD1. Using both transgenic mouse and rat SOD1-linked familial ALS (FALS) models, we found that AAK1 was partially colocalized with the endosomal and presynaptic protein markers under the normal physiological condition, but was mislocated into aggregates that contained mutant SOD1s and the neurofilament proteins in rodent models of ALS in disease. AAK1 protein levels were also decreased in ALS patients. These results suggest that dysfunction of a component in the endosomal and synaptic vesicle recycling pathway is involved in ALS pathology.

## 1. Introduction

Amyotrophic lateral sclerosis (ALS) is an adult-onset motor neuron disease characterized by predominant loss of motor neurons in the spinal cord, brain stem, and motor cortex [1]. More than 90% of ALS is sporadic (SALS) while about 20% of the familial ALS (FALS) is caused by mutations in the copper/zinc superoxide dismutase (*SOD1*) gene [2]. The availability of rodent models of ALS by overexpression of the mutant forms of SOD1 has made it possible to investigate pathogenesis of SOD1-linked ALS.

Although mechanisms of how mutations in SOD1 cause ALS remain elusive, it is evident that mutant SOD1s gain toxic properties independent of the enzyme activity that ultimately result in ALS pathology [3]. The pathological dysfunctions in ALS are multi-facet and can be summarized on two levels: Non-cell autonomous and cell death events within the motor neurons including cell surface, mitochondrial, and endoplasmic reticulum (ER) stress-mediated apoptosis, protein aggregation-related proteasome dysfunction, glutamate excitotoxicity, oxidative damage, and impaired axonal transport [4]. One underlying mechanism that is consistent with gained toxic properties of mutant SOD1s causing disease is their abilities to interact with new proteins that the wild-type (WT) SOD1 (SOD1^WT^) normally does not. In fact, these were the findings from several studies that could explain the gained toxicities. The finding that the interactions between mutant SOD1s and chromogranin A&B leading to secreted mutant SOD1s that in turn can act on microglia to cause inflammation could be responsible for the non-cell autonomous effect of the toxicity [5]. Two studies reported that mutant SOD1-specific interactions with ER-resident proteins Bip and Derlin-1 were responsible for ER stress-induced cell death [6,7]. Recently, the most comprehensive study covering a total of 132 SOD1 mutants further identified the Derlin-binding region in SOD1 that is exposed in mutant but not the WT SOD1s was responsible to interact with Derlin to cause ER stress-responses [8]. Lastly, gained interactions of misfolded mutant SOD1s with chaperones such as heat shock proteins often result in aggregate formation and reduced activities of the protein degradation pathways which can contribute to ALS pathology [9,10,11].

One of the powerful methodologies to identify interacting proteins is the yeast two-hybrid system (Y2H). To date, three studies have used this approach to have identified proteins that interacted with mutant SOD1s selectively [5,12,13]. The weakness of the Y2H systems used in those studies is of two folds: (1) The baits used did not contain a form of heterodimer consisting both the WT and mutant SOD1s that exists in naturally-occurring human SOD1-linked FALS condition; (2) The cDNA libraries used in the screenings were not from the human nervous tissues. In this study, we used a variation of Y2H known as dual bait Y2H that allowed us to improve both aspects of the screening. The dual bait Y2H allowed a copy of both the WT and mutant SOD1 to be present in yeast which will result in natural heterodimer formation between the two proteins. We also used a human brain cDNA library which is a more ALS-relevant source for protein interactions in our study. Here, we report that we have identified AAK1, an adaptor-associated kinase 1 [14] that specifically interacted with human SOD1 mutant G85R (SOD1^G85R^) in yeast. We further investigated the potential roles AAK1 might play in ALS pathology using the rodent models of SOD1-linked ALS. Our data demonstrated that in normal condition, AAK1 was located in partially overlapping locations with the endosomal and synaptic vesicle marker proteins. In ALS disease state, the patterns of AAK1 expression were altered into aggregated forms. Some of these aggregates contained mutant SOD1 proteins while others contained neurofilament proteins. In addition, the levels of AAK1 proteins were decreased in human ALS spinal cords. Taken together, our study suggests that dysfunction of AAK1 resulting in dis-regulation of endosomal and synaptic vesicle recycling pathway is likely involved in ALS pathology

## 2. Results

### 2.1. AAK1 Is Expressed in the Rodent Spinal Cord and Its Patterns of Expression Are Altered in ALS Pathology

One candidate from the duel bait Y2H screening using human SOD1^WT^ and SOD1^G85R^ as the baits was AAK1, which showed a selective interaction with SOD1^G85R^ but not with SOD1^WT^ by the yeast growth as well as the functional enzymatic activity analysis (Figure S1). However immunoprecipitation experiment using the synaptosomal preparations isolated from SOD1^G85R^ transgenic mouse spinal cord tissues did not result in detectable levels of interactions between AAK1 and SOD1^G85R^ in the mammalian content (Figure S2). This negative result was not totally unexpected and can be explained by two main factors: (i) If the interaction between AAK1 and SOD1^G85R^ in the organism is highly regulated and of transient nature, the experimental manipulation will not allow us to see such an interaction; (ii) The spinal cord clathrin-coated vesicle (CCV) preparation used in the experiment might not be the best for the detection of AAK1 and SOD1^G85R^ interaction. CCV preparations from neuromuscular junction are most relevant but technically unfeasible. It also is possible that selective interaction between AAK1 and SOD1^G85R^ does not exist in rodents. Because defect in synaptic vesicles was known to be one of the earliest event in ALS pathology [15] and the potential involvement of AAK1 in ALS pathology is completely unknown, we decided to better understand the potential role of AAK1 in ALS in general by first characterizing AAK1 expression in the spinal cord of the rodent models of SOD1-linked ALS, the knowledge of which has not been established previously.

**Figure 1:**
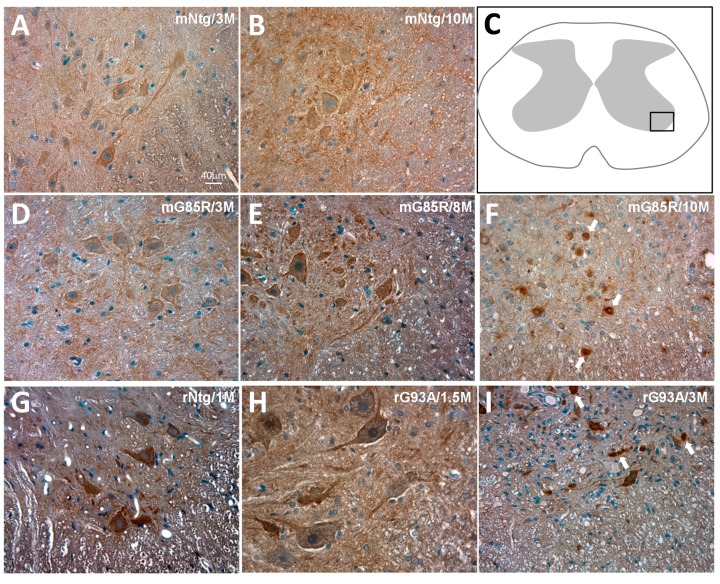
AAK1 is expressed and mislocalized into aggregates in spinal cord motor neurons in rodent SOD1-amyotrophic lateral sclerosis (ALS) models. AAK1 expression in spinal cord motor neurons was determined via immunohistochemistry which resulted in brown colored pigments. Spinal cord sections were also counter-stained with hematoxylin shown in blue. The area of the spinal cord section where the stained images are shown is indicated by the rectangular box in (**C**); Spinal cord sections were from: Non-transgenic mice (mNtg) of 3 and 10 months old (3M, 10M) (**A**,**B**); SOD1^G85R^ transgenic mice of 3, 8, 10 months old (mG85R, 3M, 8M, 10M) (**D**–**F**); Non-transgenic rat (rNtg) of 1 month old (1M) (**G**); SOD1^G93A^ transgenic rats of 1.5 and 3 month old (rG93A, 1.5M, 3M) (**H**,**I**); Some abnormal aggregated AAK1-immunoreactive appearances were indicated by the arrows (**F**,**I**). The scale bar in (**A**) applies to images (**B**) and (**D**–**I**).

Because transgenic mouse and rat models of SOD1-linked ALS with different SOD1 mutants have been well-established and both are phenotypically representative of ALS pathology [4], we chose SOD1^G85R^ transgenic mouse and SOD1^G93A^ transgenic rat lines to focus on examining the commonality of AAK1 expression in rodent spinal cords that are most relevant to ALS pathology. Immunohistochemical staining demonstrated that AAK1 was expressed in the normal, non-transgenic mouse (mNtg) as well as non-transgenic rat (rNtg) spinal cords (Figure 1A,B,G). AAK1 expression was observed in the large motor neurons which are situated in the ventral horn (part of which was illustrated in Figure 1C by the rectangular box) of the spinal cord as well as the neuronal processes with granular appearances (Figure 1A,B,G).

The onset of disease phenotype in different transgenic line of SOD1-linked ALS varies depending on the type of mutation and its level of expression. In SOD1^G85R^ transgenic mouse and SOD1^G93A^ transgenic rat line, the disease symptoms were not observed until after 10 and 3 months, respectively. We examined the pattern of AAK1 expression in the presymptomatic stages of SOD1^G85R^ transgenic mice (3M and 10M, Figure 1D,E) and SOD1^G93A^ transgenic rat (1M and 1.5M, Figure 1G,H). We observed that there was no change in AAK1 appearance in the motor neurons in the spinal cords from these animals.

However, in the end-stage diseased SOD1-linked transgenic rodent spinal cord, the AAK1 expression pattern was altered into aggregated forms (Figure 1F,I). The similar results were observed for both SOD1^G85R^ transgenic mouse and SOD1^G93A^ transgenic rat lines. This altered appearance is likely due to both loss of the motor neurons in the spinal cord as well as the formation of the potentially damaged and/or misfolded AAK1 protein aggregates (Figure 1F,I, indicated by the arrows for some aggregates).

### 2.2. AAK1 Is Partially Colocalized with the Endosomal and Presynaptic Protein Markers

Because AAK1 is known to regulate the endocytic pathway by phosphorylating the μ subunit of the clathrin-adaptor protein complexes and its expression appeared to be granular in the rodent spinal cord, we decided to determine whether AAK1 expression was in part colocalized with either endosomal and/or synaptic markers. An early endosomal marker Rab5 and a presynaptic terminal marker synaptophysin (Syn) were used for this purpose.

**Figure 2:**
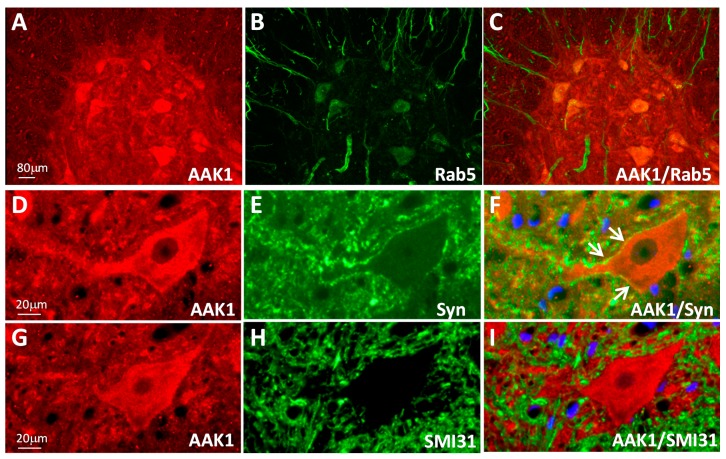
AAK1 is partially colocalized with the endosomal and presynaptic protein markers. Double immunofluorescent staining was performed using spinal cord sections for both AAK1 (red, **A**,**D**,**G**) and another protein marker (green): Rab5 (**B**), synaptophysin (Syn, **E**), and phosphorylated neurofilament protein (SMI31, **H**); The superimposed images of the two protein signals were shown in the most right panel for each section (**C**,**F**,**I**); DAPI-staining was shown in blue (**F**,**I**); Some superimposed AAK1 and synaptophysin signals on the cell surface were indicated by the arrows (**F**); Spinal cord tissues were from three month old non-transgenic mouse (**A**–**C**) and one month old non-transgenic rat (**D**–**I**). The scale bars for images (**B**,**C**) are the same as that in (**A**), for images (**E**,**F**,**H**,**I**) are the same as that in (**D**) or (**G**).

In the non-transgenic mouse spinal cord, AAK1 expression largely overlapped with that of Rab5 (Figure 2A–C). Consistent with the pattern of synaptic vesicle markers, Syn expression appeared granular and often marked the cell surface area contacted by the presynaptic terminals in both mouse and rat spinal cords (Figure S3). At a higher magnification, a partial overlap of Syn expression with that of AAK1, most notably on the cell surface of the neuron at the synapses, was observed (Figure 2D–F, indicated by the arrows). In addition, an axonal marker SMI31 which labels the phosphorylated neurofilament protein (pNF), was also included to illustrate the relative specificity of the subcellular colocalization. As expected, SMI31 labeling exhibited a complete lack of the overlapped regions with that of AAK1 (Figure 2G–I).

Therefore AAK1 is expressed in the rodent spinal cord and its pattern of expression is consistent with its known functions.

### 2.3. AAK1-Containing Aggregates Are Heterogeneous

**Figure 3:**
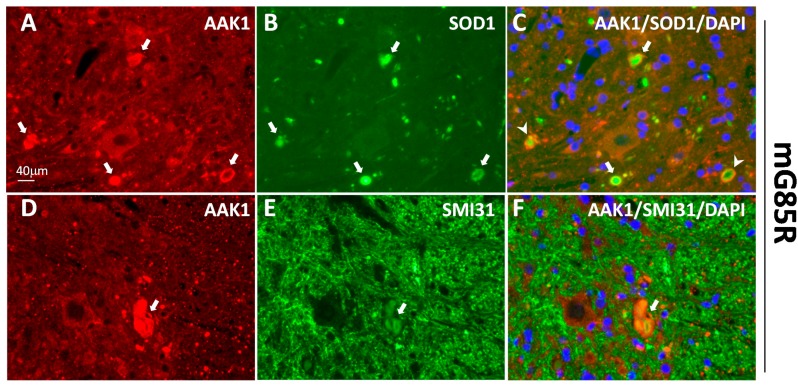

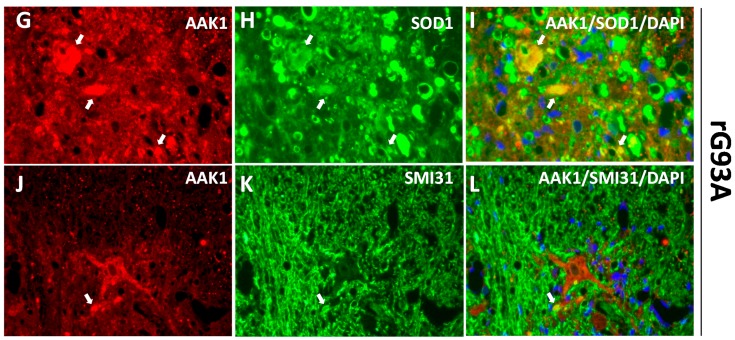
AAK1-containing aggregates are heterogeneous. Spinal cord sections from SOD1^G85R^ transgenic mouse (mG85R, 11M and end-stage, **A**–**F**) and SOD1^G93A^ transgenic rat (rG93A, 3M and end-stage, **G**–**L**) were double stained for both AAK1 (red: **A**,**D**,**G**,**J**) and another protein marker (green): mutant SOD1s (green, **B**,**H**) and phosphorylated neurofilament proteins (SMI31, **E**,**K**), via immunofluorescent-labeling. The superimposed images of the two antibody signals together with DAPI-staining (blue) were also shown (**C**,**F**,**I**,**L**). Aggregates containing both AAK1s and SOD1s or neurofilaments were indicated by the arrows (**C**,**F**,**I**,**L**) and arrowheads (**C**). The scale bar in (**A**) applies to images (**B**–**L**).

Although the stable interaction between AAK1 and SOD1^G85R^ via immunoprecipitation was not detected in the mouse spinal cord (Figure S2), the potential interactions (direct or indirect) between the two proteins were examined using double immunofluorescent labeling in symptomatic ALS animals. An antibody which selectively recognized the human SOD1 was used to demonstrate the expected appearance of aggregates containing the mutant proteins for both SOD1^G85R^ and SOD1^G93A^ animals (Figure 3B,H). When co-stained with the AAK1 antibody, some AAK1 proteins were observed in some of the SOD1^G85R^- and SOD1^G93A^-positive aggregates (Figure 3A,C,G,I indicated by the arrows). Furthermore, in a subset of SOD1^G85R^-positive aggregates, the AAK1 signal was observed to be localized more to the periphery of the aggregate as a ring structure while SOD1^G85R^ was seen more concentrated in the center of the aggregates (Figure 3C, indicated by the arrowheads). However, for the SOD1^G93A^-positive aggregates, no obvious ring-like structures of the aggregates were observed (Figure 3I).

Since not all AAK1-containing aggregates were positive for the mutant SOD1 proteins, we wanted to determine whether some AAK1 aggregates contain one of the other proteins known to be present in ALS pathology: The NF protein. Fewer AAK1-positive aggregates contained the NF proteins (Figure 3F,L, indicated by the arrows).

Taken together, AAK1 was mislocalized into aggregates which were of heterogeneous nature in rodent models of SOD1-linked ALS pathology. Some of those aggregates contained the mutant SOD1 proteins while fewer contained the NF proteins. These data suggest that the loss of AAK1 regulatory function in endosomal and potential synaptic vesicle recycling is likely involved in the general pathological events in ALS.

### 2.4. The AAK1 Protein Levels Are Decreased in Human ALS Spinal Cords

**Figure 4:**
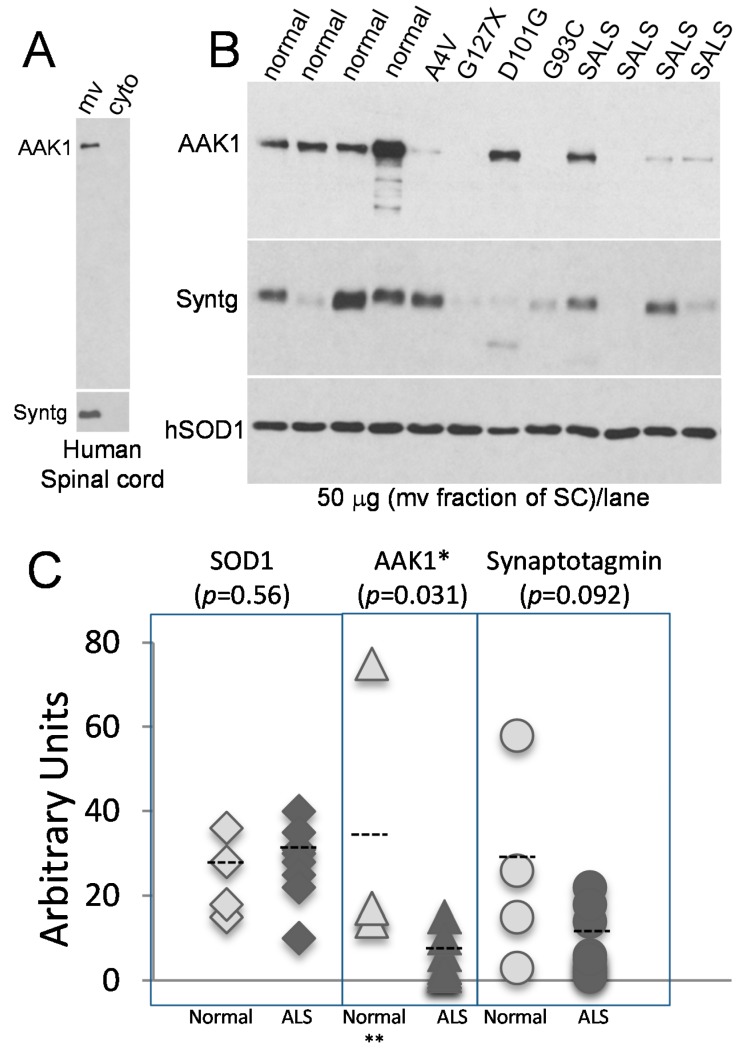
The AAK1 protein levels are significantly decreased in human ALS spinal cords. Immunoblot analysis of a total of 50 μg of proteins was used to determine AAK1 protein levels. (**A**) AAK1 proteins were enriched in the microsomal fraction. Equal amounts of proteins from the cytosolic (cyto) and microsomal (mv) fractions from the spinal cord of a normal subject were loaded for each lane. The blot was probed with both the AAK1 and synaptotagmin (Syntg) antibodies; (**B**) Microsomal proteins from both the normal (*n* = 4) and ALS (*n* = 8) spinal cords were analyzed. ALS subjects include sporadic and 4 familial cases with mutations in the *SOD1* gene (A4V, G127X, D101G, and G93C). The blots were probed with the antibodies against AAK1, synaptotagmin (Syntg), and SOD1 proteins; and (**C**) Quantified protein levels of human SOD1, AAK1 and synaptotagmin in both the normal (*n* = 4) and ALS (*n* = 8) subjects. The mean value for each group was indicated by a dotted line amongst the data points. * indicates *p* < 0.05. **: Two data points in the middle were of the same value and superimposed with each other and therefore there appears only 3 instead of 4 data points.

Lastly, to assess whether AAK1 may also be involved in human ALS pathology, we analyzed spinal cord samples from both the normal and ALS patients for the levels of AAK1 protein expression. Consistent with what’s seen in the mouse spinal cord (Figure S4), AAK1 proteins were enriched in the microsomal fraction (mv), as was the synaptic vesicle protein synaptotagmin (Figure 4A). When equal amounts of microsomal proteins were analyzed and although there were large variations in the expression amongst the human samples (Figure 4B), the AAK1 protein levels were statistically significantly lower in the ALS samples which included 4 sporadic (SALS) and 4 SOD1-linked mutations (A4V, G127X, D101G, and G93C) compared with those from the normal subjects (Figure 4C). This change was ALS-selective as the endogenous human SOD1 protein levels did not significantly differ between the two groups, nor were the levels of the synaptic vesicle protein synaptotagmin (Figure 4B,C). In the latter case, the decrease in the synaptotagmin levels in ALS did not reach statistical significance. Nonetheless, the variations seen in the synaptotagmin protein levels in the human samples were distinctively different from those seen in the AAK1 levels (Figure 4B).

The above results demonstrate that AAK1 expression is altered in human ALS pathology relative to another vesicle protein synaptotagmin.

## 3. Discussion

Although the mechanisms by which mutant SOD1s cause toxicities in ALS remain to be elucidated, there are well-established evidence to support two hypotheses: (1) The involvement of protein misfolding and aggregate formation; and (2) The impairment of synaptic vesicle recycling and presynaptic dysfunction. Our discovery of AAK1 and further study of it in the rodent models of SOD1-linked ALS suggest that AAK1 might be involved in ALS pathology via these two pathways.

### 3.1. AAK1 and Aggregate Pathology in ALS

One of the prominent features in ALS pathology was hyaline inclusions as well as neurofilament- and ubiquitin-positive aggregates in disease pathology [16,17]. Aggregates in SOD1-linked ALS have been studied extensively [18]. In the two mutant SOD1-mediated rodent ALS models examined in our study, we found that at least a portion of AAK1s in the disease state was mislocalized into aggregate-like appearances. These aggregates are of heterogeneous nature with regard to the protein components that they may contain. When either neurofilament or mutant SOD1 proteins were examined, some AAK1-positive aggregates contained neurofilament proteins and some contained either SOD1^G85R^ or SOD1^G93A^. As we do not have evidence to show direct protein-protein interactions between AAK1 and mutant SOD1 proteins, we can only assume that either they are in the same aggregates via indirect protein-protein interactions, or that general cellular stresses caused by proteins misfolding and/or decreased rates of protein degradation due to the impairment of proteasomes, lysosomes or autophagy lead to the formation of aggregates by damaged proteins including mutant SOD1s and AAK1 which are in very close proximity. Regardless of what the mechanisms might be, the existence of AAK1-positive aggregates adds one more previously unidentified protein to the list of those which have been documented before [19]. AAK1s in aggregates are likely non-functional. This result is consistent with the emerging theory that multiple pathways involving many proteins contribute to ALS pathogenesis [4].

### 3.2. AAK1 and Presynaptic Dysfunction in ALS

Shortly after the discovery of *SOD1* as the first gene to be linked to ALS, the reduction of a presynaptic marker synaptophysin in the ventral horn of ALS subjects was documented [20]. More experimental results have demonstrated the presynaptic defect caused by mutant SOD1s. The first is the finding of synaptic vesicle depletion in the neuromuscular junction as the earliest pathological event in SOD1^G93A^ mice [15]. One possible explanation for presynaptic vesicle depletion is dysfunction of synaptic vesicle recycling. Subsequently, SOD1^G85R^ mutant was also found to cause defect in the presynaptic terminal at the neuromuscular junctions measured by reduced numbers of synaptic vesicles as well as reduced intensities of the puncta staining which also correlated with the locomotor defect in *C. elegans* [21]. More recently, it was reported that SOD1^G93A^ mutant resulted in the reduced size of the synaptic vesicle pool together with the abnormal mitochondrial appearances in the presynaptic terminals in the mouse model [22].

The possibility that a general defect in vesicle trafficking is involved in ALS is further supported by the discovery of another FALS-linked gene encoding vesicle-associated membrane protein B (VAPB) associated with the late-onset form of ALS [23]. The FALS-linked mutation P56S in VAPB affected the ER structure and consequently the ER-mediated vesicle sorting and trafficking [24]. This is consistent with earlier studies to show that mutant VAPB affects endocytosis and supportive of increasing evidence demonstrating that ER stress-induced pathway also plays a role in ALS pathology in both SOD1-linked animal models and SALS [25,26,27].

The investigation of AAK1 from this study added yet another potential player that might be involved in the process leading to the defect in presynaptic terminals. Our finding that AAK1 can potentially interact with mutant SOD1 is interesting and may provide more insight into understanding ALS disease mechanisms. AAK1 was identified as a novel member of the Prk/Ark family of threonine/serine kinases that phosphorylates the μ2 subunit of the AP-2 complex as well as other proteins [14,28]. AAK1 plays an important regulatory role in clathrin-activated cargo recruitment of vesicles and the recycling of endocytosis [29,30]. The functional roles AAK1 plays in the central nervous system have not been thoroughly explored. This study is the first report to show that AAK1 is expressed in mouse and rat spinal cord motor neurons. Its presynaptic location is consistent with its known role in coating-activated receptor recruiting in vesicle endocytosis. In light of a recent publication by Watanabe *et al.* [31], which solidified the requirement of clathrin in regenerating synaptic vesicles, it is possible that AAK1 might play an important regulatory role in synaptic vesicle recycling.

Our data also showed that the abnormal distribution of AAK1 occurred after disease onset indicating that this process is not an early event in disease pathology and unlikely causative. We cannot eliminate the possibility that changes in AAK1 are consequences of the overall ALS pathology, nor can we disapprove that the regulatory function of AAK1 can be compromised earlier than the occurrence of its observable aggregated appearance which is not until symptomatic, consequently AAK1-invovled impairment in potentially regulating synaptic vesicle recycling and presynaptic function can be important in contributing to ALS pathology.

### 3.3. AAK1 and Cell Death in ALS

Our data also showed for the first time that AAK1 expression was altered in sporadic as well as multiple SOD1-linked ALS patients. The significant loss of AAK1 proteins in ALS patients is probably not unexpected as there are significant losses of axons and motor neurons where most synaptic vesicles normally reside at the end stage of ALS disease. However, the functional importance of AAK1 protein loss perhaps lies in the newly discovered role AAK1 plays in cell viability [32]. Depletion of AAK1 resulted in RKO colon carcinoma cell loss suggested that AAK1 might have additional function in apoptosis, although the mechanism by which it did so is not known. Furthermore, changes in AAK1 expression observed in Parkinson’s disease (PD) patients compared with the control subjects [33] and an intronic SNP in AAK1 found to be associated with the age of PD onset [34] suggest the potential involvement of AAK1 in PD pathology. Our study further suggests a link between AAK1 and ALS pathology, expanding its role to yet another neurodegenerative disease.

In summary, this study investigated another potential player AAK1 in ALS pathology. AAK1 was discovered by its potential interaction with mutant SOD1 protein. The mechanism by which AAK1 might be involved in ALS pathology is likely due to loss of its regulatory function in synaptic vesicle recycling which can contribute to presynaptic and ER-dysfunction as well as cell death that are known to be related to pathological events in ALS.

## 4. Materials and Methods

### 4.1. Animals

Transgenic mice expressing human SOD1^G85R^ were as originally described [35] and kindly provided by Zuoshang Xu (University of Massachusetts, Worcester, MA, USA). Transgenic rats expressing human G93A mutant (SOD1^G93A^) were described by Howland *et al.* [36]. All animal care and experiments were conducted in accordance with the guidelines established by the Institutional Animal Care and Use Committee of California Pacific Medical Center Research Institute according to Protocol #06.02.003.

### 4.2. Human Tissues

Human autopsy samples were obtained as described [37] and from The Brain and Tissue Bank for Developmental Disorders of the National Institute of Child Health and Human Development (Baltimore, MA, USA). Informed consents were obtained from all subjects before sample collections. Handlings of all human samples were in accordance with the approved protocols by California Pacific Medical Center Internal Board Review (IRB).

### 4.3. Dual Bait Yeast Two-Hybrid Screening

The yeast two-hybrid screening was performed using the Dual Bait Hybrid Hunter system (Invitrogen, Carlsbad, CA, USA) together with the cDNA library prepared from the adult human brain (Invitrogen). All procedures were performed according to the protocols provided in the Dual Bait Hybrid Hunter Instruction Manual (Invitrogen, Version C). The cDNAs of human SOD1^WT^ and SOD1^G85R^ were used as the baits.

### 4.4. Immunohistochemistry and Immunofluorescent Labeling

Transgenic mice and rats were perfused with 4% paraformaldehye in phosphate-buffered saline (PBS). The spinal cords were dissected out and fixed in the same solution for overnight at 4 °C. Tissues were further processed, embedded in paraffin, and sectioned at 7 μm thickness. Paraffin sections were processed for either immunohistochemistry using the DAB (3,3'-diaminobenzidine tetrahydrochloride, Sigma, St. Louis, MO, USA) method with the Vector ABC Kit (Vector Laboratories, Burlingame, CA, USA) or the immunofluorescent labeling. All sections were deparaffinized, incubated in 20% H_2_O_2_/10% methanol solution to quench any endogenous peroxidase activity (for the DAB method only), heat-boiled by microwaving to expose the epitopes, and blocked in 5% normal serum in PBS/0.01% Triton for 1.0 h at room temperature. Subsequently, sections were incubated with primary antibodies in PBS/1% normal serum overnight at 4 °C. For the DAB-labeling, the sections were continued for incubation with biotinylated secondary antibodies and substrates A&B. Immunoreactive signals were visualized by adding DAB for a brown color-development followed by counter-staining with hematoxylin. For the immunofluorescent labeling, the sections were continued for incubation with Cy3 or Cy5-conjugated secondary antibodies (Jackson ImmunoResearch Laboratories, West Grove, PA, USA). All images were captured on the upright Nikon microscope (ECLIPSE Ni-E, Nikon Inc., Melville, NY, USA) equipped with a CCD (charge coupled device) camera.

### 4.5. Immunoblot Analysis

Spinal cord tissues were fractionated as described [37]. The protein concentration was determined using the BCA method (PIERCE, Rockford, IL, USA). Cytosolic and microsomal proteins were separated on SDS-PAGE gels, transferred to nitrocellulose membranes, and incubated with the primary antibodies using the ECL (enhanced chemiluminescence) detection method (GE Healthcare, Piscataway, NJ, USA). The intensities of the immunoreactive signals were quantified using the ImageJ software.

### 4.6. Antibodies

The polycolonal antibody towards AAK1 was produced as described [14]. The human SOD1-specific antibody was described by Howland *et al.* [36]. Monoclonal antibody against synaptophysin was purchased from Sigma and monoclonal antibody against phosphorylated neurofilaments SMI31 was from Sternberger-Meyer Immunochemical Inc. (Jarettsville, MD, USA).

## Supplementary Materials

Supplementary figures can be found at http://www.mdpi.com/1422-0067/15/12/22918/s1.
